# Design, synthesis, crystal structure of novel hydrazone analogues as SARS-CoV-2 potent inhibitors: MD simulations, MM-GBSA, docking and ADMET studies

**DOI:** 10.1098/rsos.250373

**Published:** 2025-07-09

**Authors:** C. R. Santhosh, Sampath Chinnam, Guddekoppa S. Ananthnag, Gbolahan O. Oduselu, Kumarappan Chidambaram, Nagaraju Kerru, Nagaraju Kottam, G. M. Madhu

**Affiliations:** ^1^ Department of Chemistry, M.S. Ramaiah Institute of Technology (Affiliated to Visvesvaraya Technological University, Belgaum), Bengaluru, Karnataka 560054, India; ^2^ Department of Chemistry and BSN Center for Nanomaterials and Display, B.M.S. College of Engineering, Bull Temple Road, Bengaluru, Karnataka 560019, India; ^3^ West African Centre for Cell Biology of Infectious Pathogens (WACCBIP), University of Ghana, Accra P.O. Box LG 54, Ghana; ^4^ Department of Pharmacology, College of Pharmacy, King Khalid University, Abha, Asir Province 61421, Saudi Arabia; ^5^ Department of Chemistry, GITAM School of Sciences, GITAM University, Bengaluru, Karnataka 561203, India; ^6^ Department of Chemical Engineering, M.S. Ramaiah Institute of Technology (Affiliated to Visvesvaraya Technological University, Belgaum), Bengaluru, Karnataka 560054, India

**Keywords:** hydrazones, single crsytal XRD, ADMET, docking, MD simulations, MM-GBSA, thiohydrazone, anti-SARS

## Abstract

In this study, we have synthesized novel thiohydrazone **3(a–b)** and hydrazone analogues **3(c)** by one-pot methodology with good to excellent yields (85%–91%). *In silico* molecular dynamics (MD) simulations, docking, and absorption, distribution, metabolism, excretion and toxicity (ADMET) were evaluated as inhibitor activity against severe acute respiratory syndrome coronavirus 2 (SARS-CoV-2) (PDB ID: 5N5O) main protease. The dynamics simulations studies and docking were conducted by GROMACS and AutoDock Vina. The ADMET studies of all the compounds were evaluated to understand drug-likeness and potential for therapeutic applications. All the synthesized compounds **3(a–c)** exhibited strong docking scores between −5.0 and −5.4 kcal mol^−1^ to the targeted protein. MD simulations revealed that the **3b** protein–ligand complex demonstrated conformational stability throughout most of the 70 ns simulation period with root mean square deviation (RMSD) fluctuations below 3 Å. ADMET predictions indicated that all compounds possessed high gastrointestinal absorption, suggesting good oral bioavailability. By slow evaporation technique, crystals of compound **3b** were grown using ethanol and its single crystal X-ray diffraction analysis disclosed that it crystallized in the monoclinic (P2_1_/c) crystal system. Crystal data outlined crystal packing, bond lengths, bond angles, intermolecular hydrogen bonding parameters, etc. This study offers further avenues in the discovery of novel and promising hydrazone analogues against SARS-CoV-2 main protease.

## 1. Introduction

Coronavirus disease infections spread globally in late 2019, subsequently leading to the deaths of more than 6.8 million people globally [1,2]. It has become the deadliest virus in history. Illnesses of COVID-19 are due to severe acute respiratory syndrome coronavirus 2 (SARS-CoV-2), belonging to the *Coronaviridae* family, *Coronavirinae* subfamily*,* and genus *Betacoronavirus* [3,4]. COVID-19 symptoms include fever, cough, fatigue, chest pain, etc. Although the infections are asymptomatic, around 20% of the admitted patients required intensive care unit (ICU) facilities [5,6]. The impact of COVID-19 on human health resulted in substantial efforts from research groups to understand the virus’s structure and develop strategies to counteract the infections. It resulted in multiple promising drugs in less time, with a few marketed and some in the process of clinical trials [7,8]. However, the search for effective and safe medicines that inhibit SARS-CoV−2 continues.

SARS-CoV-2 main protease (M^pro^), PDB ID: 5N5O, is a crucial target for drug discovery as an anti-viral agent because of the essential role in viral transcription and replication. M^pro^ has a crucial role in the cleavage of viral polyproteins into functional units required for viral maturation, making it indispensable for the virus’s life cycle [9]. Unlike host proteases, M^pro^ has a unique substrate specificity, reducing the likelihood of off-target effects in humans. Its highly conserved active site across coronaviruses further enhances its appeal as a therapeutic target. Inhibiting M^pro^ effectively halts viral replication, making it a key candidate for drug discovery, aimed at developing broad-spectrum anti-viral agents.

**Figure 1.**
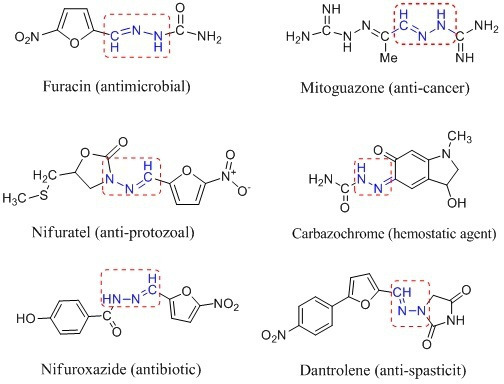
A few Food and Drug Administration (FDA)-approved and representative biomolecules containing hydrazones.

Hydrazones are a vital class of compounds in organic chemistry with the general structure R_1_R_2_C=NNH_2_ [10]. These compounds possess various biological properties, making them a vital scaffold for medicinal chemists. It includes anti-cancer [11], anti-viral [12], anti-microbial [13], analgesic [14], anti-fungal [15], anti-inflammatory [16], anti-malarial [17] and anti-tubercular activities [18,19]. Hydrazones combined with other functional groups such as thiol (-SH) and the amino (-NH_2_) can lead to compounds with distinctive physical and chemical characteristics [20,21]. Hence, the synthesis of compounds with a combination of hydrazones and other functional groups has been the research focus in recent years [22,23]. Many medications with hydrazone scaffolds have been on the market for some time. A few of them are presented in figure 1.

Several hydrazone-based derivatives were reported to be effective in the recent drug design and development for a potential SARS-CoV-2 main protease inhibition [24–26]. Modern drug discovery is supported by computational tools such as absorption, distribution, metabolism and excretion (ADME) studies, molecular dynamics (MD) studies and docking to understand drug-likeness; the interaction of ligand molecules with protein targets and protein–ligand complexes stability [27,28].

Literature reports [29] revealed that stilbenolignan analogues were the lead candidates for the inhibition of SARS M^pro^. Molecular interaction results confirmed that Gnetifolin F was competitive with a binding affinity of −8.5 kcal mol^−1^ against the targeted protein. In addition, the drug-like properties of these analogues were confirmed by absorption, distribution, metabolism, excretion and toxicity (ADMET) analysis, disclosing that stilbenolignan analogues act as potential SARS inhibitors. In another study [30], pyrazolopyrimidinone derivatives are reported for their inhibitory activity for M^pro^. Theoretical *in silico* studies, such as molecular docking and ADMET studies, confirmed their activity. Results indicated that binding energies and interaction types were comparable with those of nirmatrelvir (reference drug), and these studies confirmed the pharmacological and toxicological characteristics of the synthesized compounds. Benzimidazole derivatives synthesized from a cost-effective, green-synthetic route were disclosed to exhibit anti-SARS activity by performing docking, dynamics, and electrostatic complementarity analysis against M^pro^. Docking results indicated excellent binding scores of these compounds; protein–ligand stability was confirmed by simulation studies. This study suggests that synthesized benzimidazole derivatives are potent with considerable pharmacokinetic properties [31].

**Figure 2.**
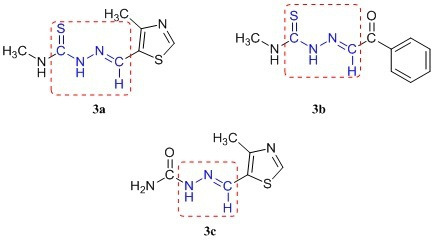
Novel thiohydrazone **3a**, **3b** and hydrazone analogues **3c** synthesized via the condensation of semicarbazides with substituted aldehydes.

**Figure 3.**
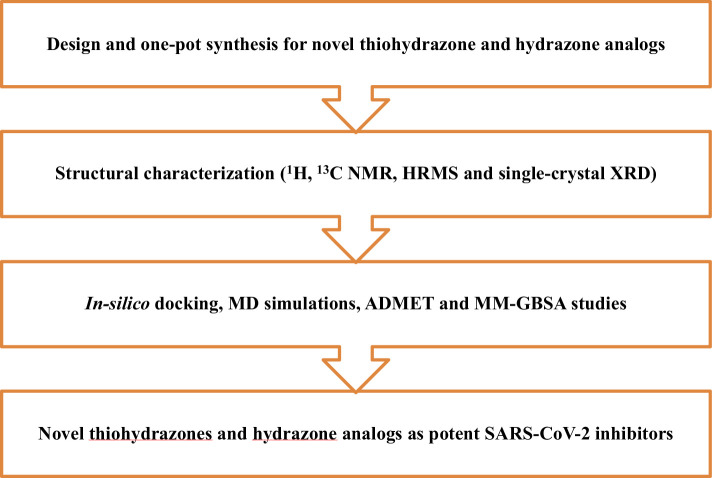
Workflow outlining the research methodology with design, synthesis, single-crystal XRD studies and computational analysis of novel thiohydrazone and hydrazone analogues as potential SARS-CoV-2 inhibitors.

For first-of-its-kind, we unveil the efficient synthetic protocol of novel thiohydrazone and hydrazone analogues (figure 2) as promising inhibitors for SARS-CoV-2 main protease. These compounds were studied for molecular docking, molecular mechanics general Born surface area (MM-GBSA), molecular dynamic simulations (MDS), and ADMET. These studies were done to get insights into the interactions, physico-chemical properties and stability of protein-receptor complexes. Single-crystal X-ray diffraction analysis was done for compound **3b** to understand various factors such as crystal packing, bond lengths, bond angles, intermolecular hydrogen bonding parameters, etc. Figure 3 illustrates the schematic overview of the research methodology employed in the current study.

## 2. Material and methods

### 2.1. Experimental

Reagents necessary for the synthesis were procured from Merck and used without being purified. All of the synthesized compounds’ melting points were measured using a Mel-Temp instrument and reported in ^o^C (uncorrected). ^1^H spectra in CDCl_3_ and DMSO-*d_6_*; ^13^C NMR spectra were recorded in CDCl_3_ and DMSO-*d_6_* by Bruker Avance 400 spectrometer. For high-resolution mass spectrometry (HRMS), ambient temperatures and Bruker microTQF-Q II ESI high-resolution were used. The chemical shifts (*δ*) were expressed in parts per million (ppm) and the coupling constants (*J*) were expressed in Hertz (Hz).

### 2.2. General procedure for the synthesis of novel thiohydrazone 3(a**−**b) and hydrazone analogues **3c**

Equimolar quantities of various semicarbazides (0.007 mol) **1(a−c)** with different aldehydes (0.007 mol) **2(a−c)** were taken into a clean 50 ml round-bottomed flask with absolute ethanol solvent, acetic acid 2−3 drops, and refluxed for 2−3 h. The reaction completion was checked by thin-layer chromatography (TLC). The solvent was removed from the resultant product using a rotary evaporator. Absolute ethanol was used for the recrystallization of the crude product. After the recrystallized solid product was obtained, it was filtered using Whatman filter paper. The novel thiohydrazone and hydrazone analogues were obtained in good to excellent yields (85%–91%).

#### 2.2.1. (*E*)-*N*-methyl−2-((4-methylthiazol−5-yl)methylene)hydrazinecarbothioamide **(3a)**

Brown solid; Yield: 87%; M.P.: 220−222 ^ο^C; ^1^H NMR (400 MHz, CDCl_3_): *δ* 10.68 (s, -NH-C( = S), 1H), 8.70 (s, -N = CH, 1H), 8.23 (s, Ar-CH, 1H), 7.33 (s, NH-CH_3_, 1H), 3.22 (d, *J* = 4.8 Hz, NH-CH_3_, 3H), 2.53 (s, Ar-CH_3_, 3H); ^13^C NMR (400 MHz, CDCl_3_): *δ* 177.7 (C = S), 155.2 (Q-C), 153.5 (imine-CH), 135.1 (Ar-CH), 127.0 (Ar-C-CH_3_), 31.2 (NH-CH_3_), 15.7 (Ar-CH_3_); HRMS of C_7_H_10_N_4_S_2_ [M+H]^+^ [*m/z*] 215.0025; Calcd: 215.0027.

#### 2.2.2. (*E*)-*N*-methyl-2-(2-oxo-2-phenylethylidene)hydrazinecarbothioamide **(3b)**

Yellow solid; Yield: 85%; M.P.: 232−234 ^ο^C; ^1^H NMR (400 MHz, CDCl_3_) : *δ* 9.77 (s, -N = CH, 1H), 7.94 (d, *J* = 7.6 Hz, Ar-CH, 2H), 7.79 (s, -NH-C = S, 1H), 7.61 (s, NH-CH_3_, 1H), 7.49−7.53 (m, *J* = 7.6 Hz, Ar-CH, 3H), 3.22 (d, *J* = 4.8 Hz, NH-CH_3_, 3H); 9.77 (s, -N = CH, 1H), 7.94 (d, *J* = 7.6 Hz, Ar-CH, 2H), 7.79 (s, -NH-C = S, 1H), 7.61 (s, NH-CH_3_ , 1H), 7.49−7.53 (m, *J* = 7.6 Hz, Ar-CH, 3H), 3.22 (d, *J* = 4.8 Hz, NH-CH_3_ , 3H); ^13^C NMR (400 MHz, CDCl_3_): *δ* 188.7 (C = O), 179.0 (C = S), 136.2 (imine-CH), 136.1 (imine-CH), 133.5 (Ar-p-CH), 129.5 (Ar-o-CH), 128.7 (Ar-m-CH), 31.6 (NH-CH_3_); HRMS of C_10_H_11_N_3_OS [M+H]^+^ [*m/z*] 222.1658; Calcd: 222.1654.

#### 2.2.3. (*E*)-2-((4-methylthiazol-5-yl)methylene)hydrazinecarboxamide **(3c)**

Yellow solid; Yield: 91%, M.P.: 248−250 ^ο^C; ^1^H NMR (400 MHz, DMSO-*d_6_*): *δ* 10.41 (s, N-NH, 1H), 9.14 (s, -N = CH, 1H), 8.18 (s, Ar-CH, 1H), 6.28 (bs, NH_2_, 2H), 2.52 (s, Ar-CH_3_, 3H); ^13^C NMR (400 MHz, DMSO-*d_6_*): *δ* 156.6 (C = O), 154.7 (imine-CH), 151.2 (Q-C), 132.8 (Ar-C-H), 129.0 (Ar-C-CH_3_), 15.2 (Ar-CH_3_); HRMS of C_6_H_8_N_4_OS; [M+H]^+^ [*m/z*] 185.0688; Calcd: 185.0687.

### 2.3. Molecular docking

Docking studies of novel thiohydrazone **3(a–b)** and hydrazone analogues **3c** were conducted with the active sites of the SARS coronavirus main protease in complex with *α*-ketoamide inhibitor (PDB ID: 5N5O). AutoDock Vina 1.1.2 via the PyRx platform was employed to do the docking analyses. The crystal structure of the main protease (SARS-CoV-2) bound to *α*-ketoamide (PDB ID: 5N5O; resolution: 2.00 Å; X-ray diffraction method) was retrieved from the Protein Data Bank (PDB). The ligands’ three-dimensional structures were generated, and energy minimization was achieved using ChemDraw Professional 15.0 [32]. The binding pockets of the protease were recognized through Chimera, based on the position of the co-crystallized *α*-ketoamide ligand. Key residues within the binding pocket included Thr190, Leu141, Phe140, Asn142, Gly143, Met49, Gln189, His164, Cys145, Ser144, Glu166, His163, Met165, His41, Asp187, His172, Thr26. The search grid for the 5N5O protein was set with coordinates (centre x: −23.158, centre y: 1.198, centre z: 2.013) and dimensions (x: 40, y: 40, z: 40) with a grid spacing of 0.375 Å. The exhaustiveness parameter was set to 8 at its default value, with all other parameters. Docking results were visually analysed using the Maestro program to identify the best binding affinities of the compounds.

### 2.4. Estimation of the molecular mechanics generalized Born surface area binding free energy

The MM-GBSA binding free energy was employed in the calculation of binding affinity using the free ligand, free receptor energy characteristics, and receptor-ligand complex [33]. The MM-GBSA estimation was performed on the docked complexes using the formula


$$\Delta {\text{G}}^{\text{bind}}={\text{G}}^{\text{complex}}-\left({\text{G}}^{\text{protein}}+{\text{G}}^{\text{ligand}}\right),$$

where ΔG^bind^ is the binding free energy of the receptor-ligand complex. G^complex^ is complex free energy, G^protein^ is the free protein energy in the absence of the ligand, and G^ligand^ is the ligand-free energy in the absence of the protein.

### 2.5. Absorption, distribution, metabolism, excretion and toxicity analysis

The compounds were analysed for ADMET properties using the ADMET lab (https://admetmesh.scbdd.com/) [34] and SwissADME (http://www.swissadme.ch/) web servers [35].

### 2.6. Molecular dynamics simulations

MD simulations in drug design and development are crucial for offering a detailed understanding of the dynamic behaviour and protein–ligand interaction stabilities. To understand the interactions of compound **3b** with SARS-CoV (PDB ID: 5N5O), an all-atom MD simulation for 100 ns was conducted [36]. This simulation was compared with that of the 8O5−5N5O complex for reference. For simulations [37], the CHARMM36 force field and GROMACS version 2022 software package were employed. The molecular topology file required for CHARMM36 was generated using the SwissParam online tool, along with the application of an explicit water model. Chloride (Cl⁻) and sodium (Na^+^) ions were added to neutralize the system. The MD protocol involved the CHARMM36 force field for energy minimization and system equilibration at 310 K within the number of particles (N), constant pressure (P), and constant temperature (T) (NPT) ensemble. The NPT ensemble was chosen to maintain constant pressure and temperature, ensuring a physiologically relevant environment. The particle mesh Ewald (PME) method, temperature held constant at 310 K, was employed to calculate long-range electrostatic interactions with barostat pressure maintained at 1 bar. A production run comprising 50 000 000 steps and 2 fs of integration time step was performed, accounting for a total simulation time of 100 ns. The selection of a 100 ns simulation time scale was based on its adequacy in capturing the dynamic stability and interactions of the protein–ligand complexes. Post-simulation analysis used XMGRACE (version 5.1.19) to visualize trajectory data. Key properties, including the hydrogen bonds (H-bonds), root mean square fluctuation (RMSF), radius of gyration (Rg), root mean square deviation (RMSD), and solvent-accessible surface area (SASA) were examined to better understand the behaviour of the protein–ligand complexes.

### 2.7. X-ray crystallography studies

Compound **3b** block-shaped crystals were allowed to grow from an ethanol solution by evaporating slowly. The fine-quality crystal, having dimensions of 0.17 × 0.11 × 0.11 mm was mounted on the Bruker D8 QUEST diffractometer. At 300 K, using Mo-Kα radiation of wavelength 1.54178 Å, the crystal was diffracted. The data were processed with SAINT and corrected for absorption using SADABS. The structure was solved using the direct method, SHELXTL, and refined by SHELXL 2013 [38]. The experimental details of diffraction are outlined in table 1.

**Table 1.** Crystallographic data and refinement parameters of structure **3b**.

chemical formula	C_10_H_11_N_3_OS
M_r_	221.28
crystal system, space group	monoclinic, P2**_1_**/c
temperature (K)	300
**a, b, c** (Å)	10.844(3), 5.8789(14), 16.429(4)
β (°)	95.637(9)
V (Å^3^)	1042.3(4)
Z	4
radiation type	Mo-Kα
µ (mm^−1^)	0.29
crystal size (mm)	0.17 × 0.11 × 0.11

data collection diffractometer: Bruker D8 QUEST diffractometer with PHOTON II detector	
absorption correction	Multi-scan—SADABS (Bruker)
T_min_, T_max_	0.656, 0.746
no. of measured, independent and observed [I > 2σ(I)] reflections	21118, 2135, 1393
R_int_	0.091
(sinθ/λ)_max_ (Å^−1^)	0.625

refinement	
R[F^2^ > 2σ(F^2^)], *wR*(F^2^), *S*	0.063, 0.118, 1.10
no. of reflections	2135
no. of parameters	140
H-atom treatment	H atoms treated by a mixture of independent and constrained refinement
Δρ_max_, Δρ_min_ (e Å^−3^)	0.25, −0.22
CCDC deposition number	24 22 211

## 3. Results and discussion

### 3.1. Chemistry

**Scheme 1.**
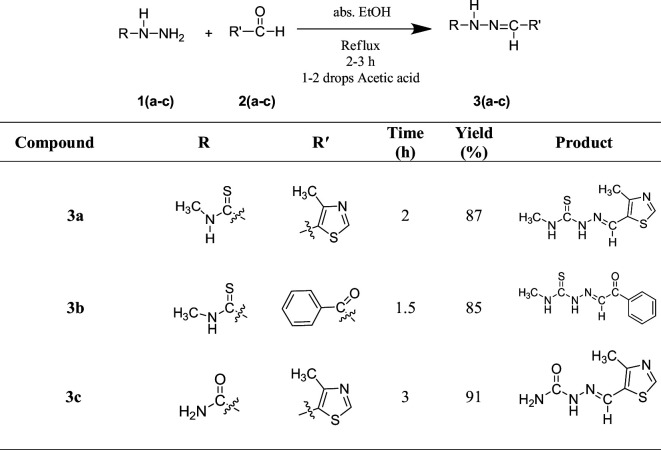
Synthesis of novel thiohydrazone **3(a–b**) and hydrazone analogues **3c**.

Novel thiohydrazone **3(a–b)** and hydrazone analogues **3(c)** were synthesized using semicarbazides, aldehydes and acetic acid (1–2 drops) in ethanol solvent. Reactants were refluxed for 2−3 h to get corresponding products **3(a–c)** (Scheme 1).

^1^H, ^13^C NMR and HRMS characterizations confirmed the structures of designed compounds **3(a–c)**. In ^1^H NMR spectra, imine (-N = CH) showed singlets as *δ* 8.70, 9.77 and 9.14 ppm for compounds **3a**, **3b** and **3c**. For –NH-C = S group, it displayed singlets as *δ* 10.68, 7.79 and 10.41 ppm for all compounds. In the case of **3a**, and **3b** compounds, NH-CH_3_ showed singlets around *δ* 7.26 and 7.64 ppm; doublets appeared for NH-CH_3_ around *δ* 3.21 and 3.23 ppm. Singlets at *δ* 2.53 and 2.52 ppm were assigned for Ar-CH_3_ and Ar-CH protons seen at *δ* 8.23 and 8.17 ppm in **3a** and **3c** compounds. In the **3b** compound, all aromatic protons appeared as doublets and multiplets at *δ* 7.94 and 7.49−7.53 ppm, whereas in compound **3c**, a broad singlet was observed for the NH_2_ group at *δ* 6.28 ppm. ^13^C NMR signals of imine (-N = CH) was shown at *δ* 153.5, 136.2 and 154.7 ppm for all compounds and other carbon signals are in good agreement with the desired compounds. Furthermore, *m/z* peaks in the HRMS spectra confirmed the molecular mass of designed compounds. The UV-visible tests were performed to check the stability of the synthesized compounds and the spectrum reports were incorporated in the supplementary file (electronic supplementary material, figures S14–S16).

### 3.2. Molecular docking analysis and molecular mechanics general Born surface area binding free energy estimation

**Figure 4.**
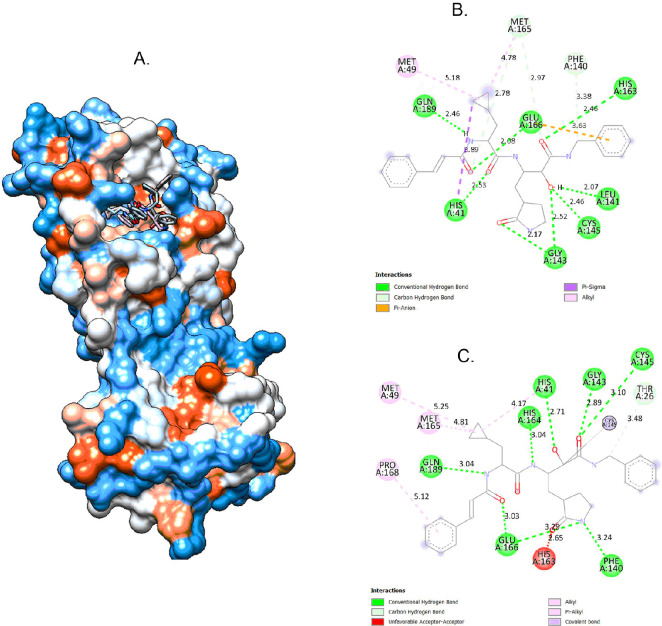
Validation of docking model (A). The protein three-dimensional structure bound to that of the docked and native co-ligands (B) docked co-ligand (C) native co-ligand.

Protocol validation for docking was achieved by the co-crystallized ligand redocking to its native position (figure 4) and comparing its interactions and RMSD from the initial pose [39]. An RMSD of 1.509 Å was observed between the native co-crystallized ligand and the docked ligand, indicating that the docking protocol successfully reproduced the native binding pose with high accuracy. Below 2.0 Å RMSD values were generally in good agreement between docked and experimentally determined poses, which suggests that the docking method is reliable. Additionally, redocking revealed that the docked ligand retained most of its initial interactions with HIS 41, GLU 166, GLN 189, MET 49 and CYS 145. The synthesized compounds were analysed for their binding affinity using molecular docking. All of the compounds showed weaker binding affinities between −5.0 and −5.4 kcal mol^−1^ with the target protein compared with Remdesivir (−7.1 kcal mol^−1^) and Hydroxychloroquine (−5.7 kcal mol^−1^) co-crystallized ligands. Their comparable docking scores to Hydroxychloroquine suggested they may still have potential for further optimization and development as anti-viral candidates.

**Figure 5.**
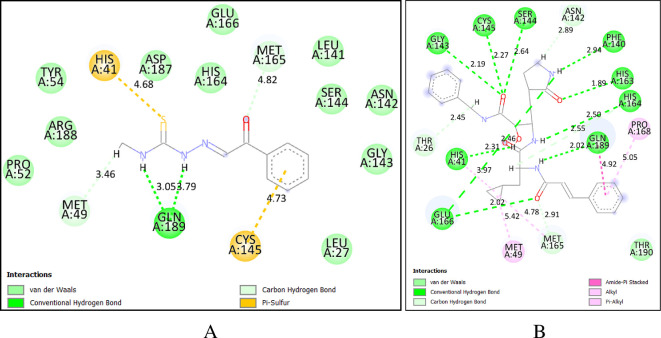
The two-dimensional interaction analysis and docking pose between the protein target and the synthesized compound **3b** along with *α*-ketoamide 8O5 (co-crystallized ligand).

All the synthesized compounds and the *α*-ketoamide 8O5 (co-crystallized ligand), Remdesivir and Hydroxychloroquine were docked to the targeted protein [40]. Two-dimensional interaction analysis and docking poses between the target protein and synthesized compound **3b,** along with *α*-ketoamide 8O5 (co-crystallized ligand) are shown in figure 5. Binding affinities and the type of interaction of these compounds are summarized in table 2. Figures depicting interactions of **3a** and **3c** along with Remdesivir and Hydroxychloroquine are included in the electronic supplementary material, figures S10–S13.

**Table 2.** Binding affinities and binding free energies of novel thiohydrazone **3(a–b)**, hydrazone analogues **3c**, *α*-ketoamide co-crystallized ligand and references (Remdesivir and Hydroxychloroquine) in the binding sites of SARS coronavirus main protease (PDB ID: 5N5O).

no.	compound	binding affinity (kcal/mol)	binding free energy (ΔGbind)	interactions (bond lengths)
1	**3a**	−5.0	−18.23	H-Bonds: GLN 189 (3.11 Å; 3.83 Å) Pi-sulfur: HIS 41 (4.68 Å), CYS 145 (5.55 Å)
2	**3b**	−5.4	−21.73	H-Bonds: GLN 189 (3.05 Å; 3.79 Å), MET 49 (3.46 Å), MET 165 (4.82 Å) Pi-sulfur: HIS 41 (4.68 Å), CYS 145 (4.73 Å)
3	**3c**	−5.1	−23.27	H-bonds: LEU 141 (5.47 Å), GLU 166 (3.31 Å), HIS 163 (5.55 Å) Alkyl/Pi-Alkyl: HIS 41 (5.73 Å), CYS 145 (4.32 Å; 7.32 Å)
4	Alpha-ketoamide 8O5 (co-crystallized ligand)	−7.3	−84.00	H-bonds: GLU 166 (2.02 Å; 2.46 Å), CYS 145 (2.27 Å), HIS 163 (1.89 Å), GLN 189 (2.02 Å), SER 144 (2.64 Å), GLY 143 (2.19 Å), PHE 140 (2.95 Å), HIS 164 (2.50 Å), HIS 41 (2.31 Å) Alkyl/ Pi-Alkyl: MET 49 (5.42 Å), PRO 168 (5.05 Å)
5	Remdesivir	−7.1	−48.22	H-bonds: HIS 163 (5.53 Å), GLN 189 (5.48 Å; 5.80 Å), PHE 140 (5.13 Å), GLU 166 (3.73 Å), GLY 143 (3.35 Å); HIS 41 (4.41 Å) Pi-Alkyl: MET 165 (4.99 Å), Pi-sulfur: CYS 145 (7.33 Å)
6	Hydroxychloroquine	−5.7	−43.83	H-bonds: GLN 189 (2.78 Å), HIS 163 (2.11 Å), GLU 166 (2.81 Å) Pi-Alkyl: MET 165 (4.86 Å), MET 49 (5.41 Å), PRO 168 (4.50 Å), HIS 41 (4.23 Å), CYS 145 (4.95 Å)

The interaction analysis of the binding of **3b** in the binding pocket of the protein revealed that it possesses a hydrogen bond interaction with GLN 189, which is one of the residues of amino acid responsible for the binding of the co-crystallized ligand to the target. Additionally, hydrophobic interactions with key residues such as CYS 145, LEU 141, MET 49, PHE 140 and MET 165 contributed to the binding affinity and stability of compound **3b** within the SARS-CoV-2 main protease active site.

The MM-GBSA module integrated with the Schrodinger suite’s prime program was employed to calculate the binding free energy (ΔGbind) of the novel thiohydrazone **3(a–b)** and hydrazone analogues **3c** and the references to the protein target. After docking analysis, the binding free energy for the compounds was calculated. **3a**, **3b**, **3c**, 8O5, Remdesivir, and Hydroxychloroquine had binding energies of −18.23, −21.73, −23.27, −84.00, −48.22 and −43.83 kcal mol^−1^, respectively, as per the MM-GBSA output (table 2). The co-crystallized ligand, 8O5 (−84.00 kcal mol^−1^) obtained the highest MM-GBSA value.

### 3.3. Molecular dynamics simulations

#### 3.3.1. Root mean square deviation

**Figure 6.**
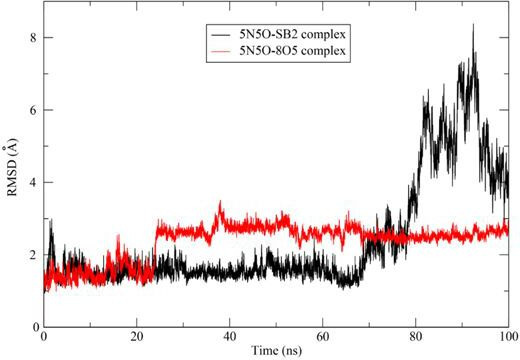
RMSD plot of the co-crystallized ligand (8O5) and the ligand **3b (SB2**) against 5N5O.

The protein–ligand complex conformational stability and fluctuations in protein MD were examined using RMSD [41]. A lower RMSD value indicated the structural stabilization of the complex. In this study, the co-crystallized ligand complex showed initial fluctuations ranging from approximately 1 to 3 Å during the 0−22 ns period, which is acceptable, and then remained stable of the remainder simulation. The **3b** complex was stable for most of the simulation period (0−70 ns), with RMSD fluctuations ranging from 1 to 3 Å. These values which are below 3 Å were within the acceptable range for small globular proteins. However, towards the end of the simulation, the fluctuations increased to over 8 Å, suggesting that the ligand **3b** may have moved away from the active site. The fluctuations of compound **3b** in MD simulations may arise from conformational flexibility, weak binding interactions or solvent effects. Interaction analysis reveals that only one hydrogen bond is formed, which could contribute to the weak binding stability of the protein–ligand complex. Figure 6 presents the RMSD plot of the co-crystallized ligand (8O5) and the ligand **3b** against 5N5O.

#### 3.3.2. Root mean square fluctuation

**Figure 7.**
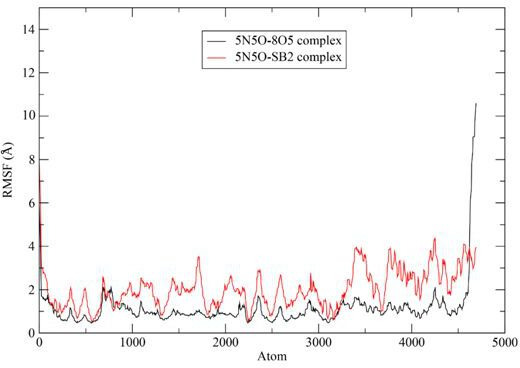
RMSF plot of the C**-**α backbone of the complexes formed between the co-crystallized ligand (8O5) and ligand **3b (SB2**) against 5N5O.

The local fluctuations of protein residues with the peaks highlighting the residues that exhibit the greatest fluctuations are understood using RMSF [42]. These peaks represent the mean deviation for each amino acid residue from its reference position. We observed significant fluctuations at the N-terminal and C-terminal regions, which is typical. In general, the co-crystallized ligand complex showed minimal fluctuations with values around 1 Å. The **3b** complex exhibited greater fluctuations than the co-crystallized ligand complex, with deviations exceeding 3 Å across most of the protein residues. The RMSF plot of the C-α backbone of the complexes formed between the co-crystallized ligand (8O5) and ligand **3b** against 5N5O is depicted in figure 7.

#### 3.3.3. Hydrogen bonds

When an electronegative atom is covalently bonded to a hydrogen atom, hydrogen bonds (H-bonds) are formed and play a pivotal role in secondary protein structures’ stability and contribute to the protein’s structural rigidity [43]. These bonds are essential for maintaining protein stability and serve as a foundation in biological systems.

**Figure 8.**
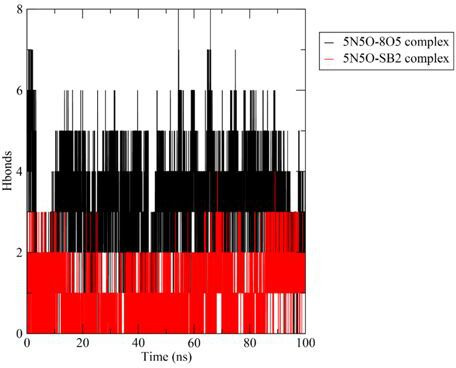
Hydrogen bonds formed between the co-crystallized ligand (8O5) and ligand **3b (SB2**) against 5N5O during the 100 ns MD simulation run.

In this study, the co-crystallized ligand complex formed as many as eight hydrogen bonds during the simulation, maintaining four hydrogen bonds for more than 90% of the simulation period. In contrast, the complex **3b** formed a maximum of four hydrogen bonds, which did not persist throughout the simulation; however, it maintained two hydrogen bonds for over 90% of the simulation period. Overall, the co-crystallized complex formed more hydrogen bonds compared with **3b**. The number of hydrogen bonds between the co-crystallized ligand (8O5) and ligand **3b** against 5N5O during the 100 ns MD simulation runs is shown in figure 8.

#### 3.3.4. Radius of gyration

Rg estimates the overall size and protein shape, helping to assess how its structure changes during MD simulations [41]. Rg provides compactness of protein and flexibility in biological environment insights, with lower values indicating a more rigid structure.

**Figure 9.**
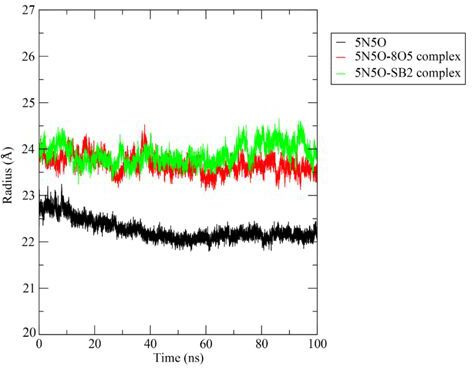
Radius of gyration observed for 100 ns dynamics simulation run for the Apo structure of the protein with the ligand **3b (SB2**) and complexed co-crystallized ligand (8O5).

In this study, the Apo structure of protein had the lowest Rg value, with fluctuations decreasing to 22 Å after 40 ns of simulation. The co-crystallized complex fluctuated around 24 Å, reaching a peak of 24.5 Å at 40 ns. The **3b** complex showed the highest fluctuations, with Rg values peaking after 50 ns, indicating greater structural variability compared with the co-crystallized ligand complex. Figure 9 presents the radius of gyration observed for a 100 ns dynamics simulation run for the Apo structure of the protein with the ligand **3b** (SB2) and complexed co-crystallized ligand (8O5) during MD simulation.

#### 3.3.5. Solvent accessible surface area

SASA is a vital factor for protein stability and folding. Importantly, it represents the area surface around a protein accessible to a solvent [44]. The molecule’s van der Waals contact surface and the hypothetical solvent sphere centre define this surface. Greater compactness of the protein indicates a lower SASA value.

**Figure 10.**
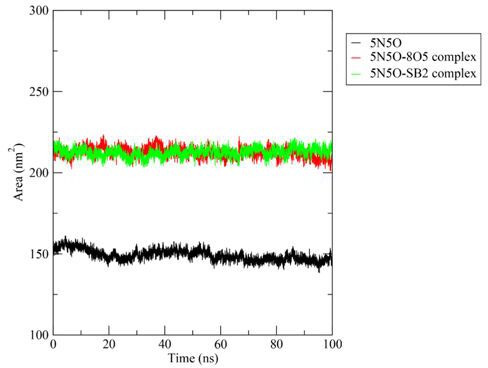
Solvent accessible surface area observed during the 100 ns dynamics simulation run for the Apo structure of the protein with the ligand **3b (SB2**) and complexed co-crystallized ligand (8O5).

The SASA results correlate with the Rg findings, where the Apo structure again had the lowest value, around 150 nm². The **3b** complex, in comparison with the co-ligand complex, had a higher SASA value towards the end of the simulation time. The SASA observed during the 100 ns dynamics simulation run for the Apo structure of the protein with the ligand **3b** (SB2) and complexed co-crystallized ligand (8O5) is shown in figure 10.

### 3.4. Structure–activity relationship studies

**Figure 11.**
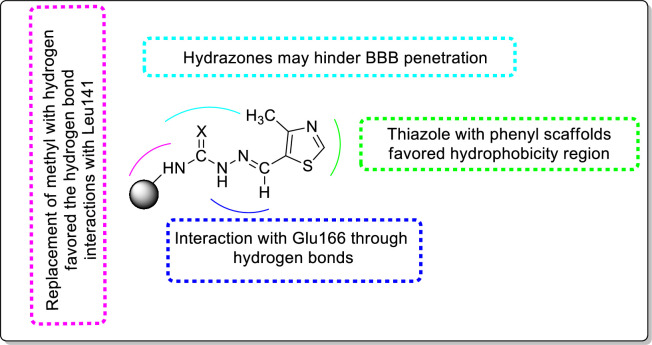
Structure–activity relationship (SAR) for the hydrazone analogues.

Structure–activity relationship (SAR) studies for hydrazone analogues are depicted in figure 11. Replacement of the phenyl moiety with its bio-isostere thiazole reduces hydrophobicity, which indicates the importance of the heterocyclic five- and six-membered rings for tuning the activity. The *pi* systems of the rings were involved in the active sites of the residues. The addition of the hydrogen of the hydrophobic methyl group on the carboxamide seemed to act as a tuner for strong hydrogen bond interactions with the Leu141 residue. These compounds displayed RMSD fluctuations ranging from 1 to 3 Å and were predicted to cross the blood–brain barrier (BBB). The designed compounds are the substrates of the Cytochrome P450 (CYP) enzyme family; particularly, CYP3a4 is crucial for drug metabolism as it regulates the biotransformation of over 90% of medications. These results indicated that novel structural skeletons of SARS-CoV-2 potent inhibitors with a good selectivity index could be suitable for further investigations.

In particular, we have chosen hydrazone analogues as potential M^pro^ inhibitors owing to their structural properties, interactions with the active site and potential mode of inhibition. They contain heteroatoms such as nitrogen, oxygen and sulfur, which can participate in hydrogen bonding and other interactions with the active site. The presence of aromatic rings in these compounds provides an opportunity for π-π stacking interactions with the active site, such as Phe140 and Tyr141. Also, heteroatoms in these compounds help in forming covalent bonds with the active site residues, thereby aiding in enzyme inhibition.

### 3.5. Absorption, distribution, metabolism, excretion and toxicity analysis

The ADMET characteristics of the compounds were evaluated to assess their ability for therapeutic usage and drug-like properties. All compounds demonstrated high gastrointestinal absorption, indicating good bioavailability through the oral route. Regarding the BBB permeability, **3a**, **3b** and **3c** compounds are predicted to be unable to cross the BBB. This may be attributed to the presence of hydrazones in **3a**, **3b** and **3c**, which may hinder BBB penetration. ADMET results of these molecules are presented in table 3.

**Table 3.** ADMET properties of designed compounds **3(a–c)**.

molecule	3a	3b	3c
Lipinski (no of violations)	0	0	0
Pgp substrate	No	No	No
GI absorption	High	High	High
CYP2C19 inhibitor	No	No	No
CYP2D6 inhibitor	No	No	No
CYP1A2 inhibitor	Yes	Yes	No
CYP2C9 inhibitor	No	No	No
CYP3a4 inhibitor	No	No	No
CL (ml min^−1^ kg^−1^)	7.766	6.894	3.96
log Kp (cm s^−1^)	−6.63	−6.21	−7.16
PAINS (number of alerts)	0	0	0
bioavailability score	0.55	0.55	0.55
BBB permeant	No	No	No
hERG blockers	0.028	0.035	0.083
DILI	0.987	0.850	0.953
Ames Toxicity	0.561	0.521	0.715

GI absorption: gastrointestinal absorption, BBB: blood brain barrier, Pgp: P-glycoprotein CYP: cytochrome P450, CL: clearance, PAINS: pan assay interference compounds, hERG: human ether-a-go-go related gene, DILI: drug-induced liver injury.

The ADMET analysis revealed that **3a** and **3b** but not **3c** are the substrates of the cytochrome P450 (CYP) enzyme family. CYP enzymes, particularly CYP3a4, are crucial for drug metabolism as they regulate the biotransformation of over 90% of medications. Specifically, **3a** and **3b,** but not **3c** were predicted to act as inhibitors of CYP1A2, a key enzyme in drug metabolism. Similarly, **3a**, **3b** and **3c** were not expected to inhibit CYP2C19 and CYP2C9, probably due to the presence of hydrazones, which seem to prevent the inhibition of these enzymes.

The Lipinski rule of 5 was used to evaluate the bioavailability of the compounds through the oral route [45,46], with no compounds violating the rule. This proposes that compounds generally comply with good oral bioavailability characteristics. Pan-assay interference compounds (PAINS) are substructures known to cause off-target effects and false positives in drug discovery [47]. In this study, none of the compounds exhibited PAINS, which is a positive indicator of their specificity and potential safety in further testing. Regarding clearance, all compounds are predicted to have moderate clearance of 5–15 ml min^−1^ kg^−1^. Thus, it suggests that the compounds would be eliminated at a reasonable rate, which is important for maintaining therapeutic levels in the body. The human ether-a-go-go related gene (hERG), drug-induced liver injury (DILI), Ames (mutagenicity) toxicity potentials were also predicted for the compounds. The hERG predictions suggest a low risk of cardiotoxicity, while the DILI scores indicate a high likelihood of liver toxicity, necessitating further *in vitro* and *in vivo* evaluations to confirm hepatotoxic risks. These toxicity predictions suggest moderate mutagenic potential, which may require structural modifications or additional screening to minimize genotoxic risks.

### 3.6. X-ray crystallography studies

**Figure 12.**
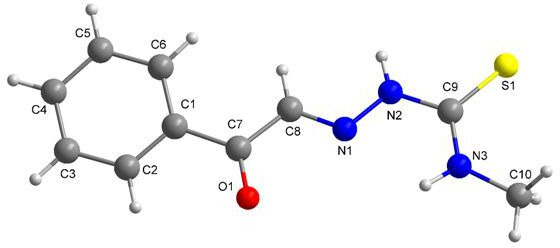
Molecular structure of **3b.**

**Figure 13.**
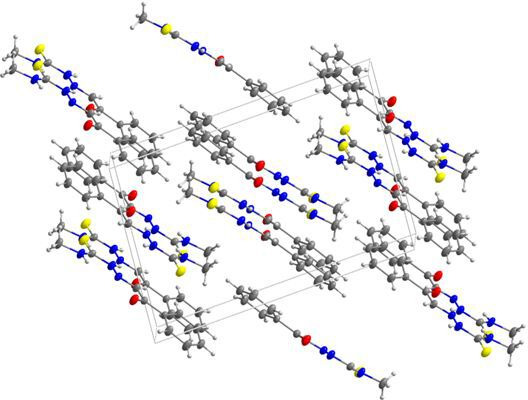
Crystal packing diagram in the unit cell of **3b.**

Crystals of compound **3b** were grown from an ethanol solution by slow evaporation. Mounted fine-quality crystal was diffracted using Bruker D8 QUEST diffractometer (Mo-Kα radiation of wavelength 1.54178 Å at 300 K). Crystal structure was determined by the direct method with refined by SHELXL 2013 and SHELXTL [38]. The molecular structure of **3b** and the numbering of the atoms scheme are shown in figure 12. The crystal packing diagram is depicted in figure 13. The angles and bond lengths selected are listed in table 4. The crystal exists in the monoclinic crystal system. The C9 = S1 and C8–N1 bond distances in compound **3b** are 1.661(3) and 1.270(3) Å, respectively. The bond lengths are comparable to similar thiasemicarbazide derivatives in literature [48–50]. Intermolecular hydrogen bonds between N2–H2 and O1^i^ and N3–H3 and S^ii^ exist in the molecule. Further, an intramolecular hydrogen bonding between N3–H3 and N1 is also observed. The hydrogen bond geometry details are presented in table 5.

**Table 4.** Selected bond lengths/angles of compound **3b** (Å,°).

bond lengths (Å)		bond angles (°)	
C1–C7	1.489(3)	C8–N1–N2	117.2(2)
C7–C8	1.477(4)	N1–N2–C9	120.1(2)
O1–C7	1.215(3)	C9–N3–C10	123.3(2)
N1–C8	1.270(3)	O1–C7–C1	121.3(3)
N1–N2	1.349(3)	C8–C7–C1	117.5(2)
N2–C9	1.367(3)	O1–C7–C8	121.2(2)
S1–C9	1.661(3)	N1–C8–C7	119.0(3)
N3–C9	1.314(3)	N2–C9–S1	119.9(2)
N3–C10	1.450(3)	N3–C9–N2	115.8(2)
		N3–C9–S1	124.4(2)

**Table 5.** Hydrogen-bond geometry in compound **3b**.

D–H···A	D–H	H···A	D···A	D–H···A
N2–H2···O1i	0.86	2.29	3.091(3)	154.4
N3–H3···S1ii	0.89(3)	2.67(3)	3.303(3)	129(3)
N3–H3···N1	0.89(3)	2.18(3)	2.616(3)	110(3)
symmetry codes: (i) x, y−1, z; (ii) x, y+1, z.				

CCDC deposition number: 24 22 211.

## 4. Conclusion

In this study, novel thiohydrazone **3(a–b)** and hydrazone analogues **3c** were synthesized and characterized as viable SARS-CoV-2 main protease inhibitors. The current study has important advantages such as excellent yields, shorter reaction times, simple work-up procedure, single crystal X-ray structure and potent SARS-CoV-2 inhibitors. These compounds were evaluated to understand their drug-likeness and physico-chemical characteristics via ADMET, molecular docking, followed by dynamics simulations and MM-GBSA calculations. Among the synthesized compounds, **3b** demonstrated the most promising binding affinity (−5.4 kcal mol^−1^) and stability in MD simulations, indicating its ability as a lead compound for future optimizations. However, its binding affinity was lower compared with standard inhibitors such as Hydroxychloroquine −5.7 (kcal mol^−1^), Remdesivir (−7.1 kcal mol^−1^) and the co-crystallized *α*-ketoamide ligand (−7.3 kcal mol^−1^). Molecular docking analyses revealed significant interactions with key residues (e.g. GLU 166, CYS 145, MET 165) within the main protease active sites of SARS-CoV-2. Furthermore, MD simulations validated the stability of these interactions over a 100 ns time scale, with RMSD, RMSF, hydrogen bond analysis, Rg and SASA assessments indicating that **3b** maintained a stable interaction with the protein, despite some fluctuations towards the later stages of the simulation. Despite the moderate docking and MD results, these results highlight the potential of the hydrazone scaffold as a prospective starting point for the development of SARS-CoV-2 inhibitors. The compound **3b** crystal structure was determined by single-crystal X-ray diffraction analysis. Crystal data gave insights into hydrogen bonding parameters and their arrangements within the molecule. Future studies should focus on structural modifications of these hydrazone analogues to enhance their binding affinities and drug-like properties. *In vivo* and *in vitro* analyses are required as a part of the experimental validation of these compounds to understand their anti-viral activity and assess their safety profiles. Moreover, DFT study with FMOs including HOMO and LUMO gaps could be the subject of our future studies. These results contribute to the research on hydrazone analogues and their significant role in counteracting COVID-19.

## Data Availability

All data are available in the main text or the electronic supplementary material [51]. Crystallographic data of the compound **3b** has been deposited at the Cambridge Crystallographic Data Centre under the reference number CCDC−2422211.
